# Comment on “Effect of Health Education on Willingness to Undergo HIV Screening among Antenatal Attendees in a Teaching Hospital in North Central Nigeria”

**DOI:** 10.1155/2016/2959608

**Published:** 2016-09-14

**Authors:** Siddharudha Shivalli, Soujanya Kaup

**Affiliations:** ^1^Department of Community Medicine, Yenepoya Medical College, Yenepoya University, Mangalore, Karnataka 575018, India; ^2^Department of Ophthalmology, Yenepoya Medical College, Yenepoya University, Mangalore, Karnataka 575018, India

Sekoni et al. [1] have investigated the effect of health education on willingness to undergo HIV screening among antenatal attendees in a teaching hospital in North Central Nigeria. In their quasi-experimental study, authors concluded that health education is a strategy to enhance voluntary counseling and testing uptake in antenatal settings. However, the following issues and concerns need to be addressed.

The authors state that a minimum sample size of 122 was estimated using the formula by Kirkwood for the comparison of two proportions. The authors should have considered attrition while calculating the sample size. In addition, authors should have included a CONSORT flow diagram for the study participants [2].

In Table  3 of the article [1], *p* value for the study group is reported as *p* = 0.00000. Practically, the value of *p* cannot be zero and, hence, we would suggest to report it as *p* < 0.0001. Also in Table  3, chi-square (*χ* ^2^) calculation for the control group is questionable as at least 20% of expected frequencies are less than 5. Did the authors consider Yates's or any other apt correction for chi-square (*χ* ^2^)? Authors should have clearly addressed this in methodology section. Similar mistakes are reflected in Table  6 [1].

In Tables  5 and 6 of the article [1], authors have represented before and after intragroup comparisons. However, the purpose of having a control group in the study is to do an intergroup comparison and they should have done that. At the baseline, both study groups should be comparable with no significant differences in the demographic and other key study variables. The following points are noted on intergroup comparison in this study:

(i) Baseline awareness of HIV/AIDS among the intervention group (*n* = 120, 99.2%) was similar to that among control group (*n* = 113, 97.4%) (*χ* ^2^ = 1.11, *p* = 0.293).
(ii) Baseline perceived benefits in knowing HIV status among the intervention (*n* = 116, 95.9%) and control (*n* = 114, 98.3%) groups were similar (*χ* ^2^ = 1.11, *p* = 0.273).
(iii) Baseline willingness to know the HIV status was significantly higher (*χ* ^2^ = 4.53, *p* = 0.033) among the controls (*n* = 103, 88.8%) when compared to the intervention group (*n* = 95, 78.5%).
(iv) On the contrary, at the baseline, significantly lower proportion of controls knew about the HIV testing facility in the hospital (35.3% versus 65.3%, *p* < 0.001).

The knowledge of availability of HIV testing in the hospital is an effect modifier in this study. These two factors can explain 2% decline in willingness to know the HIV status on end line survey in the control group. In the intervention group, on the contrary, a significant increase in the proportion of participants willing to test for HIV is attributed to awareness of availability of testing facility and relatively lower level of willingness to undergo HIV testing at the baseline. An adjusted analysis for the effect modifier and differences in the baseline study variables by applying logistic regression would have been a better option to see the actual effect of the intervention. Addressing all the afore-discussed factors is necessary to yield unbiased results.

The debated issues here are minor criticisms and are unlikely to modify the results. Nonetheless, authors must be congratulated for investigating an important public health issue.
